# Clinicopathological features of primary thyroid Burkitt’s lymphoma: a systematic review and meta-analysis

**DOI:** 10.1186/s13000-020-00933-z

**Published:** 2020-02-08

**Authors:** Toshitetsu Hayashi, Mitsuyoshi Hirokawa, Seiji Kuma, Miyoko Higuchi, Ayana Suzuki, Risa Kanematsu, Takumi Kudo, Naomi Katsuki, Reiji Haba, Akira Miyauchi

**Affiliations:** 1 Department of Diagnostic Pathology and Cytology, Kuma Hospital, 8-2-35 Shimoyamate-Dori, Chuo-Ku, Kobe, Hyogo 650-0011 Japan; 2 Department of Internal Medicine, Kuma Hospital, Kobe, Hyogo Japan; 3 Department of Diagnostic Pathology, Takamatsu Red Cross Hospital, Takamatsu, Kagawa Japan; 4 Department of Diagnostic Pathology, Faculty of Medicine, Kagawa University, Takamatsu, Kagawa Japan; 5 Department of Surgery, Kuma Hospital, Kobe, Hyogo Japan

**Keywords:** Thyroid Burkitt’s lymphoma, Hashimoto’s thyroiditis, Meta-analysis, Clinicopathological features

## Abstract

**Background:**

Primary thyroid Burkitt’s lymphoma (BL) is an extremely rare and highly aggressive form of non-Hodgkin’s lymphoma; only isolated case reports are available for patients with this disease.

**Methods:**

We analyzed the clinicopathological features of thyroid BL by conducting a meta-analysis of 21 known patients (including ours) and compared them to those of extrathyroidal BL.

**Results:**

There were 13 men and 8 women with a median age of 39.3 years (range, 6–75 years). The median follow-up was 46.5 months (range, 0.5–361 months). Six patients (28.6%) had stage I disease, 2 (9.5%) had stage II, 2 (9.5%) had stage III, and 11 (52.4%) had stage IV. Five of 7 tested patients with thyroid BL (71.4%) had histological evidence of underlying Hashimoto’s thyroiditis. Ki-67 labeling indices exceeding 90% in all 19 patients tested (100%). Fluorescence in situ hybridization performed on 12 patient samples revealed that all (100%) had *MYC* rearrangement. Among the 16 patients for whom follow-up data were available, 4 died of disease-related causes. Kaplan-Meier analysis revealed that the 12- and 60-month overall survival rates for patients with thyroid BL were 87.5 and 70.7%, respectively.

**Conclusions:**

Ours was the largest study of thyroid BL and its detailed clinicopathological features to date. Thyroid BL is not associated with underlying Epstein-Barr virus infection but is closely linked to Hashimoto’s thyroiditis; patients generally have good overall survival and respond well to intensive chemotherapy. The correct pathological diagnosis is essential for treatment selection and outcome improvement.

## Background

Primary thyroid Burkitt’s lymphoma (BL) is a rare and highly aggressive form of non-Hodgkin’s lymphoma and comprises 1–2% of thyroid lymphomas [1]. This neoplasm is characterized by intermediate-sized lymphoid cells with a “starry sky” appearance and exhibits chromosomal translocations that activate the *MYC* oncogene [2, 3]. To our knowledge, only 20 patients with primary thyroid BL have been described in the English-language literature [4–16]. Because of its extremely low prevalence, little is known about the pathogenesis and clinicopathological features of this disease, or about the differences between it and its systemic counterpart.

We report a new patient with primary thyroid BL to help further characterize the clinicopathological and genetic features of this disease. We also performed a meta-analysis of all 21 patients known to date and compared their characteristics to those of patients with extrathyroidal BL.

## Methods

### Research design

We reviewed 454 patients diagnosed with primary thyroid lymphoma at Kuma Hospital, Kagawa University, and Takamatsu Red Cross Hospital between 1996 and 2015. One patient with BL (0.2%) was identified according to the following diagnostic criteria: 1) monomorphic medium-sized cells with basophilic cytoplasm, 2) “starry sky” appearance, 3) positive expression of CD20 and CD79a, and 4) extremely high Ki-67 labeling index [17]. B cell lymphomas with intermediate features that spanned those of diffuse large B cell lymphoma and BL were excluded. The clinical data were obtained from electronic medical records.

Immunohistochemical studies were conducted on an automated stainer (Ventana-Biotech, Tucson, AZ) using formalin-fixed, paraffin-embedded materials. The following antibodies were used: CD3 (clone 2GV6, Ventana-Biotech; dilution 1:100), CD5 (clone SP19, Ventana-Biotech; dilution 1:100), CD10 (clone SP19, Ventana-Biotech; dilution 1:100), CD20 (clone SP67, Ventana-Biotech; dilution 1:100), CD21 (clone 2G9, Ventana-Biotech; dilution 1:100), CD30 (clone BerH2, Ventana-Biotech; dilution 1:30), CD43 (clone L60, Ventana-Biotech; dilution 1:200), CD79a (clone SP18, Ventana-Biotech; dilution 1:200), IgD (rabbit, polyclonal, Ventana-Biotech; dilution 1:100), IgG (polyclonal, Ventana-Biotech; dilution 1:100), IgA (polyclonal, Ventana-Biotech; dilution 1:100), IgM (polyclonal, Ventana-Biotech; dilution 1:100), Kappa (polyclonal, Ventana-Biotech; dilution 1:100), Lambda (polyclonal, Ventana-Biotech; dilution 1:100), Bcl-2 (clone SP66, Ventana-Biotech; dilution 1:100), Bcl-6 (clone GI191E/A8, Ventana-Biotech; dilution 1:100), MUM-1 (clone MRQ-43, Ventana-Biotech; dilution 1:100), p16 (clone EP1551Y, Abcam, Cambridge, UK; dilution 1:100), p53 (clone DO7, DAKO, dilution 1:1000), Ki-67 (clone 30–9, Ventana-Biotech; dilution 1:100), and MDM2 (clone 2A10, Abcam; dilution 1:100). Diffuse reactivity was defined as labeling of ≥30% of the tumor cells, focal reactivity as 1–29% labeling, and no reactivity as negative staining.

Analysis using Epstein-Barr virus (EBV)-encoded small RNA (EBER1 and EBER2) fluorescein-conjugated EBER peptide nucleic acid probe (DAKO PNA ISH Detection Kit, K 5201) was performed. The appearance of brown color in the nucleus was considered a positive reaction. Tissue from a patient with nasopharyngeal carcinoma who was known to be positive for EBV was used as a positive control in each run. *MYC*/*IgH* detection by fluorescence in situ hybridization (FISH) (commercial MYC/IGH/CEP8 set) was also performed.

### Search strategy and meta-analysis

Comprehensive searches restricted to English-language documents were conducted. We searched for articles listed in the PubMed (Public/Publisher Medline) database up to December 2018 using the terms “thyroid”, “Burkitt’s lymphoma”, and “primary”; the initial search retrieved 44 publications. We used the following inclusion criteria: 1) human patients, 2) relevant histopathological and/or immunohistochemical findings, 3) apparent clinical outcome and treatment effects, and 4) defined as lymphoma involving either the thyroid gland alone or the thyroid gland and adjacent neck lymph nodes without contiguous spread or distant metastases at the time of diagnosis, whereupon 32 articles were excluded. Hence, we reviewed 12 articles that described 20 patients with thyroid BL and also included our own patient in the meta-analysis.

### Statistical analysis

Overall survival was calculated from the date of diagnosis until that of the last follow-up. Data regarding the literature review were extracted from the corresponding articles. Survival curves were estimated according to the Kaplan-Meier method, and disease-free survival was calculated from the date of progression to that of the last follow-up. The analysis and graphs were obtained using STATFLEX version 6 (Artech Co., Ltd., Osaka, Japan).

## Case presentation

A 49-year-old woman with a history of Hashimoto’s thyroiditis presented with a rapidly growing neck mass and upper airway compression symptoms. No B symptoms were present. The hematological test showed a white blood cell count within the normal range (51.5 × 10^2^/μL). Interleukin-2 receptor (801 U/mL) and lactate dehydrogenase (228 U/L) levels were elevated, although serum thyroxine and thyroid-stimulating hormone levels were within their normal ranges. An elevated thyroid peroxidase antibody of 271.4 IU/mL was detected. Ultrasonography revealed a mass measuring 2.0 × 1.0 × 1.0 cm in the right lobe of the thyroid; the border of the nodule was indistinct. The patient underwent a right thyroidectomy after a core needle biopsy revealed a diffuse large B-cell lymphoma. Following the final histological diagnosis of primary thyroid BL, the patient received rituximab, cyclosphosphamide, doxorubicin, vincristine, and prednisone for 2 weeks, as well as rituximab, hyperfractionated cyclophosphamide, vincristine, doxorubicin, and dexamethasone including prophylactic intrathecal methotrexate for 3 months. Adjuvant treatments involving 22 cycles of linear accelerator (LINAC) therapy, 40 Gy each, were administered after the chemotherapy. Thirty months after the initial diagnosis, the patient had no evidence of recurrent disease.

### Microscopic findings

**Fig. 1:**
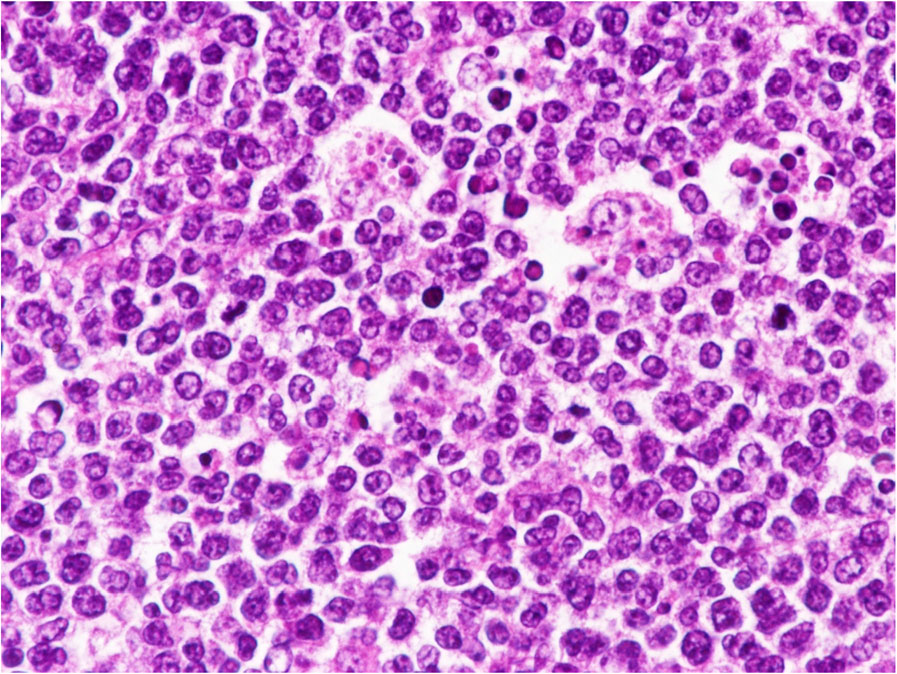
Histopathological findings of thyroid Burkitt’s lymphoma. The tumor cells are composed of round, intermediate-sized cells with round nuclei admixed with scattered tingible body macrophages imparting a “starry sky” appearance (hematoxylin and eosin, × 400)

The tumor cells were composed of round, intermediate-sized lymphoid cells admixed with scattered tingible body macrophages imparting a “starry sky” appearance (Fig. 1). The nuclei were uniform and round-to-oval-shaped. The chromatin was coarsely clumped and had medium-sized paracentral nucleoli, while the cytoplasm was basophilic. Mitotic figures (12 per high-power field) were identified; there was no coagulative necrosis, although extrathyroidal extension was observed. Hashimoto’s thyroiditis was identified in the non-tumoral thyroid tissue.

### Immunophenotypic and cytogenetic findings

**Fig. 2:**
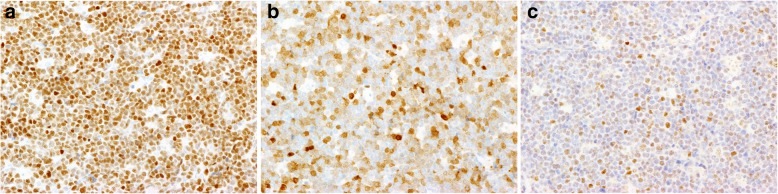
Immunohistochemical staining of thyroid Burkitt’s lymphoma: The nuclei are positive for MUM-1 (**a**) and p16 (**b**), and focally positive for MDM2 (**c**) (× 200)

**Fig. 3:**
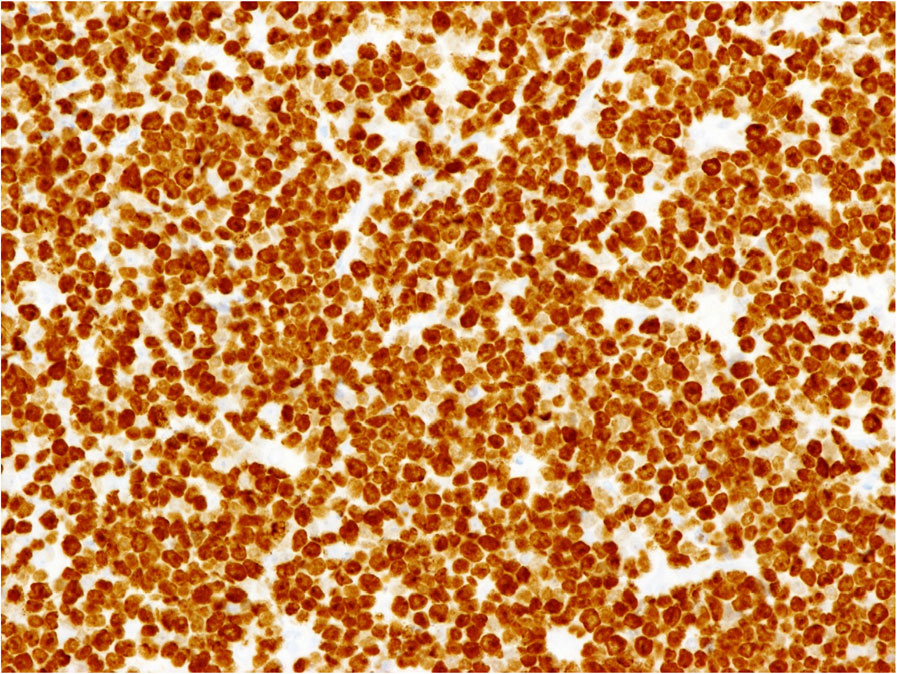
Immunohistochemical staining of thyroid Burkitt’s lymphoma. Almost all of the tumor cells are immunoreactive against Ki-67 (× 200)

**Fig. 4:**
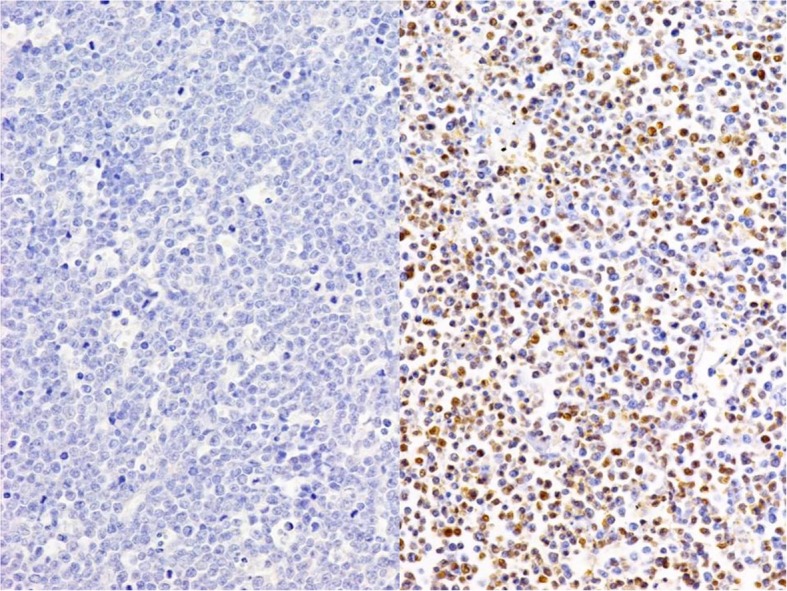
Left: Epstein-Barr virus-encoded small RNA (EBER) in situ hybridization of thyroid Burkitt’s lymphoma. The tumor cells are negative for Epstein-Barr virus (EBV) (×200). Right: EBER in situ hybridization of nasopharyngeal carcinoma, which was used as external positive control. The tumor cells are positive for EBV (× 200)

**Fig. 5:**
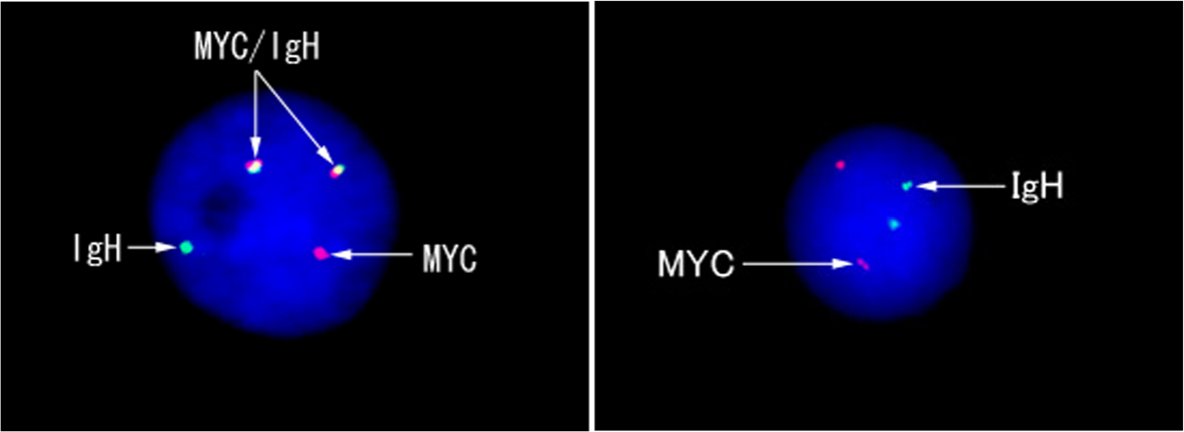
MYC/IgH fluorescence in situ hybridization of thyroid Burkitt’s lymphoma. Left: The MYC/IgH translocation is demonstrated with 1 separate green signal, 1 separate red signal, and 2 fused green/red signals on the tumor cell. Right: Normal human lymphocyte as a control indicated by 2 red and 2 green signals

Diffuse membranous immunostaining for CD10, CD20, and CD79α, as well as diffuse nuclear staining for MUM-1 (Fig. 2a) and p16 (Fig. 2b), were noted in the tumor cells. The expression of MDM2 was focal and confined to the tumor cell nuclei (Fig. 2c), although the cells were negative for Bcl-2, Bcl-6, and p53. The Ki-67 labeling index exceeded 90% (Fig. 3). The EBER in situ hybridization test was negative (Fig. 4). Ninety-six percent of the tumor cells were found to have *MYC*/*IgH* gene fusion as determined by FISH (Fig. 5).

#### Meta-analysis

##### Clinical findings

The clinicopathological features of 21 thyroid BLs are summarized in Table 1. The median age at diagnosis was 39.3 years with a male-to-female ratio of 13/8. The majority (94.4%) of these patients presented with a thyroid mass; patients also presented with a rapidly growing nodule (66.7%), dyspnea (61.1%), dysphagia (16.7%), and thyrotoxicosis (5.6%). Four of the 14 described patients (28.6%) showed B symptoms (e.g., systematic symptoms of fever, night sweats, or weight loss). None of the reported patients presented with immunosuppressive conditions or the endemic form of BL. Regarding the treatment modalities, all of the 20 patients for whom data were available (data for 1 were missing) received multidrug chemotherapy regimens; 12 received chemotherapy only, 7 received chemotherapy with combined surgery, and 1 was administered chemotherapy combined with surgery and adjuvant radiotherapy (LINAC).

**Table 1:** Clinicopathological characteristics of 21 thyroid Burkitt’s lymphomas by meta-analysis of the literature

Age (years)	6–75 (median 39.3)
Sex (Male/Female)	13/8
Clinical presentation	
Thyroid mass	17/18 (94.4%)
Rapid growing	12/18 (66.7%)
Dyspnea	11/18 (61.1%)
Dysphagia	3/18 (16.7%)
Thyrotoxicosis	1/18 (5.6%)
Cavernous sinus syndrome	1/18 (5.6%)
*B symptoms	4/14 (28.6%)
Associated immuosuppressive condition	0/21 (0%)
Tumor size (mm) (median size)	20–105 (59)
Other organ involvement at diagnosis	11/21 (52.4%)
Lymph nodes involvement	11/21 (52.4%)
Above diaphragm	7/11 (63.6%)
Above and below diaphragm	4/11 (36.4%)
Clinical stage at presentation	
I	6/21 (28.6%)
II	2/21 (9.5%)
III	2/21 (9.5%)
IV	11/21 (52.4%)
Elevated thyroid function	2/8 (25%)
Elevated TSH	1/2 (50%)
Elevated thyroglobulin	1/2 (50%)
Elevated thyroid antibody	4/8 (50%)
Hashimoto’s thyroiditis	5/7 (71.4%)
Immunohistochemistry	
CD3	0/7(0%)
CD5	0/8(0%)
CD10	14/14 (100%);
CD20 (*n* = 18)	18/18 (100%)
CD21 (*n* = 2)	0 (0%)
CD30 (*n* = 2)	0 (0%)
CD43 (*n* = 3)	1/3 (33.3%)
CD79a (*n* = 5)	5/5 (100%)
TDT (*n* = 3)	0 (0%)
IgG (*n* = 1)	0 (0%)
IgA (*n* = 1)	0 (0%)
IgM (*n* = 1)	1/1 (100%)
Kappa (*n* = 2)	1/2 (50%)
Lambda (*n* = 1)	1/1 (100%)
Bcl-2 (*n* = 10)	0 (0%)
Bcl −6 (*n* = 6)	5/6 (83.3%)
MUM-1 (*n* = 2)	1/2 (50%)
p16 (*n* = 1)	1/1 (100%)
p53 (*n* = 1)	0/1 (0%)
Ki-67 labelling index (> 90%)	19/19 (100%)
MDM2 (*n* = 1)	1/1 (100%)
EBER ISH	0/12 (0%)
Translocation t(8;14) (*n* = 12)	12/12 (100%)
CD10 + (Flow cytometry)	5/5 (100%)
Treatment	
Chemotherapy only	12/20 (60%)
Surgery + Chemotherapy	7/20 (35%)
Surgery + Chemotherapy + Radiotherapy	1/20 (5%)
Follow-up (months) (median)	0.5–361(46.5 m)
Clinical outcome	
Alive with complete remission	14/19 (73.7%)
Alive with persistent disease	1/19 (5.3%)
Dead of disease	4/19 (21.1%)

*EBER ISH* Epstein - Barr virus Small RNAs In Situ *Hybridization, FISH fluorescence* in situ *hybridization, LINAC* Linear particle accelerator therapy

^*^B symptoms: systemic symptoms of fever, night sweats, or weight loss

##### Histological, immunohistochemical, and cytogenetic findings

Five of 7 patients with thyroid BL (71.4%) had morphologic evidence of Hashimoto’s thyroiditis. Regarding immunohistochemical features, all patients who were tested for CD10 (*n* = 14), CD20 (*n* = 18), and CD79a (*n* = 5) were positive for these respective proteins. IgM was positive in the lone patient who was tested. Additional factors measured are shown in Table 1. The Ki-67 labeling index exceeded 90% in all 19 patients for whom such staining was performed. We demonstrated that our patient had focal immunoreactivity for MDM2, but none of the other patients had been tested for this protein. All 12 patients who were tested for EBER in situ hybridization showed negative results, and flow cytometry immunophenotyping demonstrated CD10-positive monotypic B-cell populations in all 5 tested patients; moreover, *MYC* gene translocation was detected using FISH in all 12 patients whose samples were tested.

##### Survival

**Fig. 6:**
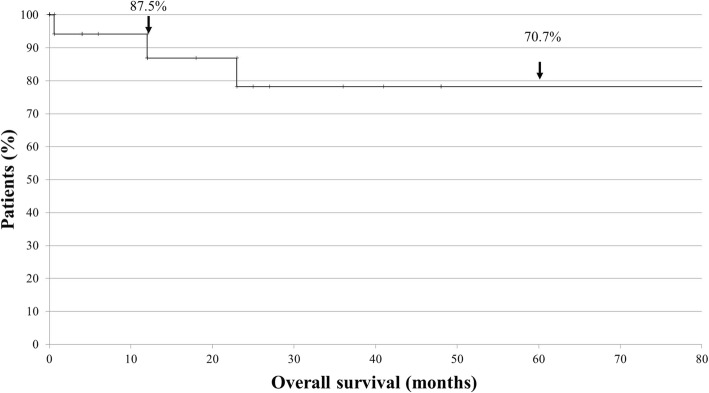
Kaplan-Meier curves showing the overall survival of 16 patients with thyroid Burkitt’s lymphoma. The 12-month and 60-month overall survival rates were 87.5 and 70.7%, respectively

Of 19 patients with available clinical outcome data, 14 (73.7%) were alive with complete remission, 1 (5.3%) was alive with persistent disease, and 4 (21.1%) died of the disease. Three of the 19 patients lacked follow-up data; hence, the median follow-up of the 16 remaining patients was 46.5 months (range, 0.5–361 months). Kaplan-Meier survival analysis showed that the 12- and 60-month overall survival rates were 87.5 and 70.7%, respectively (Fig. 6).

## Discussion and conclusions

Although BL is well understood and extensively studied at the clinical and molecular levels [2, 3, 18–20], thyroid BL remains incompletely characterized owing to its extremely low incidence rate. There are no extensive case series of patients with thyroid BL, as only sporadic reports exist to date. As such, the pathogenesis, clinicopathological features, and optimal treatment methods for thyroid BL have not been well established.

The present systematic review and meta-analysis of patients with thyroid BL demonstrated that this entity mostly occurs in adult patients (the median age was 39.3 years) with a male predominance (the male-to-female ratio was 13:8). Based on our data, the most common presenting symptom is a thyroid mass, followed by a rapidly growing nodule, dyspnea, dysphagia, and thyrotoxicosis.

Histologically, thyroid BL is composed of intermediate-sized lymphoid cells with monomorphic nuclei admixed with scattered tingible body macrophages imparting a “starry sky” appearance. Regarding the immunohistochemical results, the vast majority of thyroid BLs have a late germinal center that is positive for CD10 (100%) and Bcl-6 (83.3%). Bcl-2 was negative in all patients tested for it. The proliferation rate as determined by the Ki-67 labeling index approached 100%. Moreover, *MYC*/*IgH* translocation was present in all 12 examined patients.

The current 2016 World Health Organization classification defines BL as a highly aggressive lymphoid neoplasm that frequently occurs at extranodal sites or presents as acute leukemia [17].

Tumors comprise monomorphic medium-sized B cells with basophilic cytoplasm and high mitotic rates. A range of histological characteristics has been observed in patients with BL, and the previously described categories of ‘atypical BL’ and ‘Burkitt-like lymphoma’ have since been eliminated [17]. The tumor cells have a late germinal center phenotype positive for CD10 and Bcl-6 but negative for Bcl-2 and Tdt. The Ki-67 labeling index is extremely high. On the molecular level, a reciprocal translocation involving the *MYC* oncogene on chromosome 8 at band 8q24 is a mainstay of this type of disease. According to the WHO classification, the diagnosis of BL should be a combination of morphology, genetic, and immunophenotype. BL is defined as a c-MYC single hit lymphoma, additional molecular abnormal defied this diagnosis. Except for the result of c-MYC rearrangement, if possible, the result of Bcl-2 and Bcl-6 rearrangement should be detected [17]. In our case, both Bcl-2 and Bcl-6 are negative by immunohistochemistry. As such, our meta-analysis suggests that there are no substantial differences in morphology, immunophenotype, and genetic features between thyroidal versus extrathyroidal BL.

Extrathyroidal BL has 3 variants, endemic, sporadic, and immunodeficiency-related, that are similar in morphologic, immunophenotypic, and genetic features [21, 22]. A recent gene expression profiling study demonstrated that the endemic and immunodeficiency-related BL variants have an almost identical molecular profile, whereas the profile of sporadic BL is distinct [21]. Endemic BL is associated with malaria and EBV, and frequently involves the jaws or orbits [21], whereas sporadic BL is less frequently associated with EBV infection (although EBV is detected in approximately 30% of patients with this variant) and frequently involves the abdominal cavity, especially the ileocecal region [22, 23]. Immunodeficiency-related BL is more often encountered in patients with HIV infection/AIDS or, less frequently, in subjects with congenital or iatrogenic immunodeficiency. This variant tends to involve the lymph nodes and extranodal sites such as the bone marrow and gastrointestinal tract [22]. To our knowledge, endemic or immunodeficiency-associated BL has not been described in the thyroid, and the results of our meta-analysis also indicate that thyroid BL constitutes a distinct subgroup of EBV-negative sporadic BL that is closely associated with Hashimoto’s thyroiditis.

The prototypical immunophenotypic presentation of EBV-negative BL is a higher MUM-1 expression [24], lower prevalence of p53 overexpression [22], and a late germinal center immunophenotype (MUM-1+/Bcl-6+) when compared to EBV-positive BLs (43, 46.2, and 40% vs. 14, 30, and 21%, respectively). MUM-1 is considered an immunohistochemical marker of the late germinal center immunophenotype and post-germinal center B cell, and the morphologic spectrum of MUM-1-positive cells ranges from that of a centrocyte to that of a plasmablast/plasma cell. On the other hand, Bcl-6 expression is observed immediately after the B cell enters the germinal center and is maintained only until the germinal center exit. Unlike most normal germinal center B cells in which the expression of MUM1 and Bcl-6 are mutually exclusive, BL tumor cells show co-expression of MUM-1 and Bcl-6, suggesting that these markers are dysregulated in BL [24]. The lower prevalence of p53 overexpression is frequently observed in EBV-negative extrathyroidal BL [19, 20, 25]. Auto-regulatory factors such as MDM2 (which inactivates p53) or p16 are found in a variety of human tumor tissues [26] and BL cell lines [19]. Although the number of investigated patients in our meta-analysis is small, the concurrence of a lack of p53 immunoreactivity with MDM2 and p16 positivity suggests that p16 involvement and p53-MDM2 pathway alterations may also occur in thyroid BL in a manner similar to that of EBV-negative BL [19].

To date, the outcomes of patients with thyroidal and extrathyroidal BL have not been compared. Extrathyroidal BL is uncommon in adults, where it has a worse prognosis [27]. Previous studies reported a 60-month overall survival rate of 46% in patients with extrathyroidal BL, as well as a complete remission rate of 20% post-chemotherapy [28]. In our meta-analysis, thyroid BL was associated with a higher 60-month overall survival (70.7%) and a good overall response to chemotherapy with high rates of complete remission (74.7%).

From a practical standpoint, the distinction between thyroid BL and other high-grade B cell neoplasms is of major clinical importance given that the treatment regimens for BL and diffuse large B cell lymphomas differ substantially [29]. Thyroid BLs may be treated very effectively with highly intensive chemotherapy and central nervous system prophylaxis such as intrathecal methotrexate, as well as adequate supportive care such as the prevention of tumor lysis syndrome; these interventions lead to excellent overall survival rates among these patients [4, 30]. Therefore, adequate and prompt pathological diagnosis is essential for treatment selection and outcome improvement for patients with thyroid BL.

The principal strength of our study was the integration of the information obtained from a high number of publications related to thyroid BL as well as incorporating our own experience with this disease. Our findings particularly contribute to better understanding the clinicopathological features and optimizing the management of thyroid BL. The limitation of the study was its retrospective nature as a systematic review and meta-analysis; hence, the data were susceptible to reporting biases and to tailored study eligibility criteria. Despite this limitation, we used strict inclusion criteria and statistical methods to examine this extremely rare disease to provide new insights. Additional multicenter prospective studies are required to further clarify the clinicopathological features of thyroid BL.

This is the largest study that defines the detailed clinicopathological features of thyroid BL to date. Although thyroid BL has overlapping morphological and immunophenotypical features with extrathyroidal BL, we highlighted the fact that the former constitutes a subgroup of EBV-negative sporadic BLs with epidemiologic and clinical features as well as pathogenetic mechanisms that are distinct from the latter. Patients with thyroid BL also have much better overall survival and favorable response rates to intensive chemotherapy than do those with extrathyroidal BL. Adequate pathological diagnosis is essential for treatment planning and favorable outcomes for patients with this disease.

## Data Availability

All data generated or analyzed during this study are included in this published article and its supplementary information files.

## Abbreviations

BL: Burkitt’s lymphoma
EBER: Epstein-Barr virus-encoded small RNA
EBV: Epstein-Barr virus
FISH: fluorescence in situ hybridization
LINAC: Linear particle accelerator therapy
